# A Method for Sensing Dielectric Properties of Thin and Flexible Conductive Biocomposites

**DOI:** 10.3390/s24113508

**Published:** 2024-05-29

**Authors:** Andrea Cataldo, Christian Demitri, Leonardo Lamanna, Antonio Masciullo, Raissa Schiavoni

**Affiliations:** Department of Engineering for Innovation, University of Salento, 73100 Lecce, Italy; christian.demitri@unisalento.it (C.D.); leonardo.lamanna@unisalento.it (L.L.); antonio.masciullo@unisalento.it (A.M.); raissa.schiavoni@unisalento.it (R.S.)

**Keywords:** conductive biocomposites, dielectric properties, dielectric permittivity, microwave reflectometry, electromagnetic simulations, open ended coaxial probe

## Abstract

This study investigates the dielectric properties of conductive biocomposites (CBs), which are integral to the development of advanced materials for flexible electronics and medical devices. A novel method employing Microwave Reflectometry (MR) is introduced, utilizing a miniaturized Vector Network Analyzer (m-VNA) and a dedicated sensing element (SE), to extract the dielectric properties of CBs. The method is grounded in a minimization principle, aligning the measured $S_{11}$ reflection scattering parameter with its electromagnetic (EM) simulation, facilitating a refined process for determining the dielectric properties. The experimental setup was meticulously engineered, optimized, and validated using reference dielectric samples (RDSs) with known dielectric properties. The method was then applied to three innovative CBs, resulting in an accurate extrapolation of their dielectric properties. The findings highlight the method’s versatility, cost-efficiency, and applicability to ultra-thin and flexible biopolymer films, offering significant potential for advancements in flexible electronics and bio-sensing applications.

## 1. Introduction

Biocomposites are a class of polymers that are produced by biological processes [1,2] involving natural sources such as proteins [3], nucleic acids [4], and polysaccharides [5]. Consequently, their structure and function vary based on the living organisms from which they originate. In this context, it is possible to group biocomposites into three broad categories: *polynucleotides* composed by nucleotides of RNA and DNA, *polypeptides* made up of proteins and shorter polymers of amino acids, and *polysaccharides* primarily consisting of sugary carbohydrates like cellulose [6,7]. Nowadays, biocomposites are used in a large number of applications, and this success is mainly due to their biodegradability characteristics [8], which effectively minimize the impact that human activities have on the environment [9]. As an additional advantage, biocomposites can be replenished over time since they are made of materials derived from renewable sources [10]. For all these reasons, biocomposites represent the key materials for the future, as they are gaining prominence in various fields, mainly in medicine [11,12] and the food industry [13]. For instance, biocomposites can be useful in developing medical implant organs [14], drug delivery systems [15], tissue scaffolds and tissue engineering [16,17,18], or for food packaging purposes [19].

The incorporation of conductive fillers [20,21] into polymer matrices allows for the production of conductive biocomposites (CBs). This approach is gaining attention as an eco-friendly alternative [22] to conventional conductive materials, which are often non-renewable and environmentally detrimental. Specifically, CBs can find applications in bioelectronics [23], as wearable or implantable sensors [24], due to their combination of biological and electrical properties and, more importantly, their compatibility with living human tissues. In addition, CBs offer benefits in terms of diverse chemical composition, easily customizable form and structure [25], and flexibility [26]. On the other hand, CBs exhibit mechanical characteristics that are distinct from those found in commercially widespread polymers, resulting in them being more fragile [27]. As a result, various dissimilar material components have been combined into CBs to overcome mechanical limitations and enhance conductivity and long-term stability properties. In this context, one promising approach is the use of carbon fibers or graphite as conductive filler [28]. This study utilized a matrix of ethyl cellulose—a biopolymer derived from cellulose, the most abundant biopolymer in nature—and plasticized with vegetable oil. [29]. A thorough sensing of the dielectric properties of CBs is imperative to fully understand and optimize the performance of these composite materials. However, nowadays, fully understanding their dielectric capabilities remains a challenge.

In this regard, state-of-the-art methods mainly include various spectroscopy techniques, based on the analysis of the CBs’ response to an external electric field. Some of the best-known techniques include electrical impedance spectroscopy (EIS) [30], and scanning probe microscopy (SPM) [31,32]. However, these methods present several disadvantages since spectroscopy-based techniques strongly depend on the sample’s opacity or thickness, and often require complex modeling to interpret the data correctly. On the other hand, SPM techniques, such as atomic force microscopy (AFM) [33], provide high-resolution surface characterization but can be invasive and potentially alter the sample’s properties. Moreover, the characterization and measurement of ultra-thin CB films present additional challenges related to the thinness of CBs that could be damaged during the measurement phase. These limitations underscore the need for improved methods that can offer non-invasive, accurate, and reliable sensing of the dielectric properties of CBs, including in their thin film configuration. In this regard, this work presents a novel method for the sensing of the dielectric properties of CBs, useful to fully understand and customize their electrical properties. This method also offers new perspectives for advanced applications, ensuring optimal performance and reliability. To achieve these objectives, a Microwave Reflectometry (MR)-based system, coupled with a customized sensing element (SE), has been developed to analyze the CB electromagnetic (EM) response to a stimulus. Notably, the MR technique, as implemented in the proposed approach, is non-invasive, thus preserving the sample’s integrity, which is particularly crucial for ultra-thin CBs [34]. In addition, it can provide accurate and consistent measurements, eliminating the need for complex data interpretation. In this work, the adoption of this technique was combined with the development of a minimization procedure useful to extrapolate the frequency-dependent dielectric properties of CBs. This objective is achieved by comparing the obtained measured reflection scattering parameter ($S_{11,real}$) with the simulated one ($S_{11,mod}$), derived from a validated model obtained through the use of a full wave EM simulator. This MR-based system is adaptable to various CB types and configurations and also shows advantages in terms of versatility and cost-effectiveness.

The paper is structured as follows. Section 2 reports the theoretical background of the MR technique, details the experimental setup, and describes the CBs developed in this study. Section 3 outlines the proposed method, delving into the validation process of the simulated model and the adopted minimization procedure. Finally, Section 4 reports the results obtained by applying the proposed method on test CBs. Conclusions and future work are outlined in Section 5.

## 2. Foundations and Materials

The subsequent discussion delves into the theoretical aspects of MR techniques, discusses the experimental setup, and introduces the innovative CBs developed in this research.

### 2.1. Microwave Reflectometry and Dielectric Properties

Microwave Reflectometry (MR) is a versatile technique used for various monitoring and diagnostic applications in numerous fields, including non-destructive evaluation [35,36], medical diagnostics [37,38,39], geophysical investigation [40,41,42], and remote sensing. This method is advantageous for its cost-effectiveness, prompt feedback, the capability for remote control, and high measurement accuracy. Specifically, in MR, a low-power EM test signal is sent to the material under test (MUT). This signal is reflected from the MUT due to EM interactions, carrying useful information about the MUT’s dielectric properties. To interpret this information, specialized data-processing algorithms are employed to analyze the so-called reflection scattering parameter ($S_{11}\left(f\right)$), expressed in magnitude and phase. This allows us to obtain an estimate of the dielectric permittivity of the MUT. In more detail, the dielectric permittivity is considered a complex function of the frequency and it can be expressed by the following equation:

(1) $$\varepsilon^{*}\left(f\right)=\varepsilon^{\prime }\left(f\right)-j\varepsilon^{\prime \prime }\left(f\right)$$

where *j* is the imaginary unit while $\varepsilon^{\prime }\left(f\right)$ and $\varepsilon^{\prime \prime }\left(f\right)$ are the real and the imaginary parts of complex dielectric permittivity, respectively.

### 2.2. Experimental Setup

**Figure 1:**
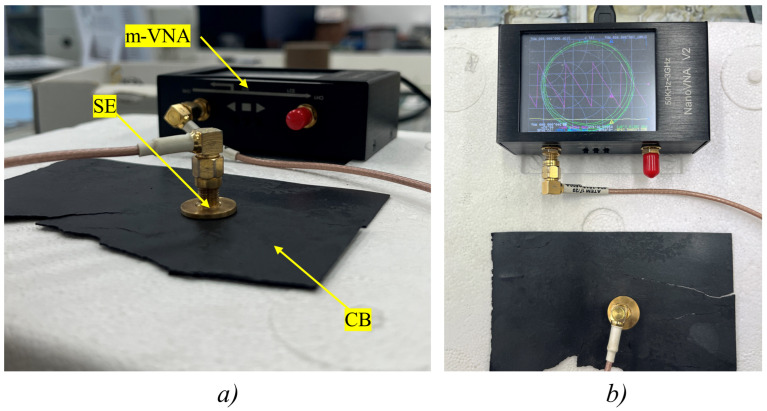
Experimental setup for dielectric property sensing of CBs using the MR technique. The custom-designed SE is in direct contact with the conductive CB under test and connected to the m-VNA, which measures the reflected signal and aids in the accurate determination of the CBs’ dielectric properties. Cross-sectional view (**a**). Top view (**b**).

In this study, MR measurements were performed through a miniaturized Vector Network Analyzer (m-VNA); the VNA was developed by HCXQS and operates in a frequency range of 50 kHz–3 GHz with a dynamic range of over 90 dB. It was adopted in combination with a sensing element (SE) properly designed for the present application. More in detail, an m-VNA has several considerable advantages over classic benchtop VNAs in terms of reduced cost (about 100 euros), portability, reduced size, simpler functionality, and a good cost-to-performance ratio with good metrological performance [38]. These characteristics serve as a foundation for the development of the innovative procedure for non-destructive biocomposite film characterization, which represents the main novelty of this work. On the other hand, a specific SE was developed based on the open truncated coaxial probe configuration [43,44]. It should be emphasized that a punctual sensing is advantageous. It enables the precise targeting of specific sample surface areas, facilitating a detailed assessment of the dielectric characteristics’ uniformity. Given that biocomposites are typically produced through specialized processes known to introduce variations in homogeneity, this targeted approach is particularly beneficial. Specifically, the inner conductor has an outer diameter measuring 1.25 mm, while the outer conductor has an inner diameter of 4.2 mm. Teflon serves as the dielectric material situated between the two conductors. The probe has a length of 11 mm. It is designed to achieve a depth of the sensing volume in the order of 0.5 mm, specifically tailored to facilitate the analysis of ultra-thin CBs in the frequency range of the instrument without compromising their structural integrity. To achieve the optimal configuration, extensive electromagnetic simulations were conducted in CST Microwave Studio^®^. In addition, a flange with a diameter of 17.2 mm was integrated into the SE to capitalize on the fringing effect, thereby enhancing the electric field’s interaction with CBs and extending the sensing capabilities beyond the physical boundaries of the probe. The experimental setup is shown in Figure 1.

### 2.3. Conductive Biocomposites

**Figure 2:**
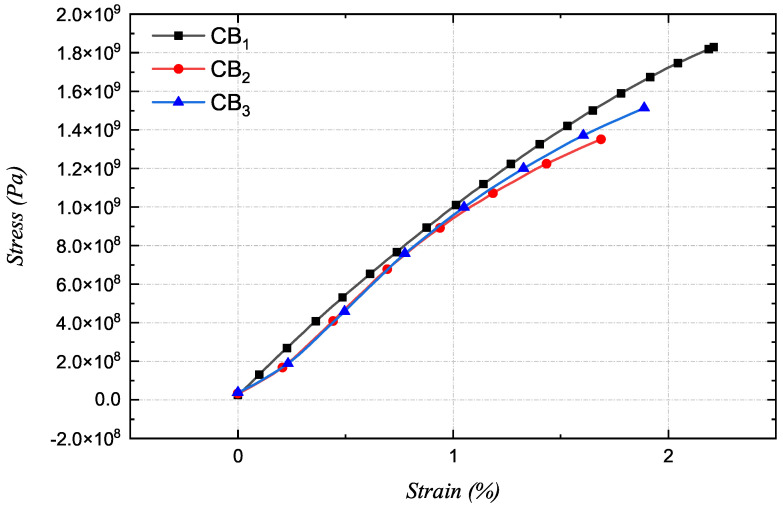
Tensile stress–strain measurement of CBs.

This study highlights the sustainability and non-toxicity of the preparation process, aiming for a scalable solution-based approach using ethanol as a green solvent. The material presented builds upon a previous formulation developed by the research group [45]. The biocomposites were prepared by dissolving ethyl cellulose (48–49.5% *w*/*w* ethoxyl basis), soybean oil, and dispersing the conductive filler in ethanol. The solution was agitated for 24 h. Afterward, the dispersion was cast onto a petri dish to dry at room temperature, with ethanol as the solvent. The ethanol completely evaporated during the drying process, leaving behind the final material. This method ensures that no solvent residues remain in the material, resulting in a clean and controlled casting process. The process produces films with a thickness of 500 µm, with a margin of error of 5 µm. A 5% concentration of conductive filler, relative to the matrix, was utilized, proving sufficient for forming a continuous conductive network. Three prototypes were developed and subsequently tested to evaluate their dielectric properties. The first prototype, $CB_{1}$, incorporated carbon fibers as the conductive filler. The second, $CB_{2}$, used expanded graphite. The third, $CB_{3}$, underwent a sonication process after the 24-h mixing period to enhance the dispersion of the expanded graphite in the solution. Subsequently, tensile tests were carried out to characterize the CBs and evaluate several mechanical figures of merit, such as elastic modulus, stress, and strain at break, as reported in Figure 2. The materials exhibit an elastic modulus ranging from 1.2 GPa to 900 MPa, with strain at break between 1.5% and 2.2% and maximum sustained stress consistently exceeding 1 GPa. Interestingly, the sample $CB_{3}$, which underwent a sonication process to enhance the material’s electrical properties, also showed improved mechanical properties. Compared to the non-sonicated $CB_{2}$, $CB_{3}$ demonstrated an increase in both stress and strain at break. This improvement is attributed to the better dispersion of expanded graphite, which enhances load dissipation.

## 3. Proposed Method

**Figure 3:**
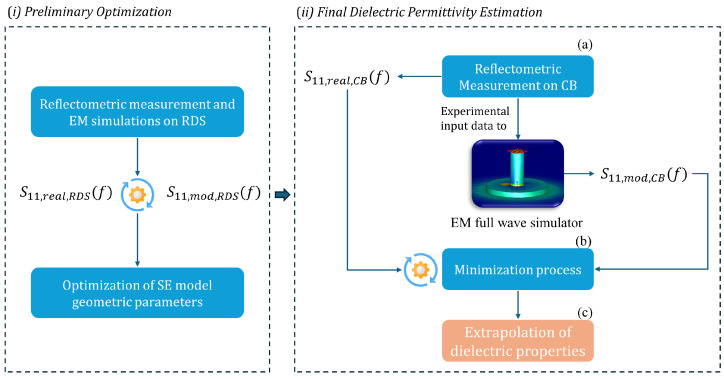
Pipeline of the proposed method for the extrapolation of CBs’ dielectric properties.

As previously mentioned, an innovative technique for extracting dielectric properties from CBs has been developed. To facilitate comprehension of the proposed method, a schematic representation detailing the principal steps is presented in Figure 3. As shown in step (i), the method first involves the validation process of the simulated model using reference dielectric samples (RDSs) with known dielectric characteristics. Specifically, this stage is crucial to ensure that the setup model is as representative as possible of the actual model. In this way, it was possible to compare the system’s simulated response with actual measurements in terms of $S_{11}\left(f\right)$ to optimize the geometric properties of the modeled SE. The method proceeds with step (ii), which consists of the final minimization procedure, executed within the EM simulator, useful to extrapolate the dielectric properties of CBs. This goal is achieved by employing the parameterization of dielectric properties of CBs to minimize the gap between simulated and measured data. More details on the two phases are given below.

### 3.1. Preliminary Optimization on Reference Dielectric Samples

The experimental tests and related simulations were conducted by placing the SE in contact with a thin slot filled with RDSs (respectively, methanol, ethanol, and propanol) whose frequency-dependent dielectric properties are extrapolated from the National Physical Laboratory (NPL) report [46]. The slot was set within a polystyrene block since its dielectric properties are very close to air, in order to minimize interference due to other materials.

**Figure 4:**
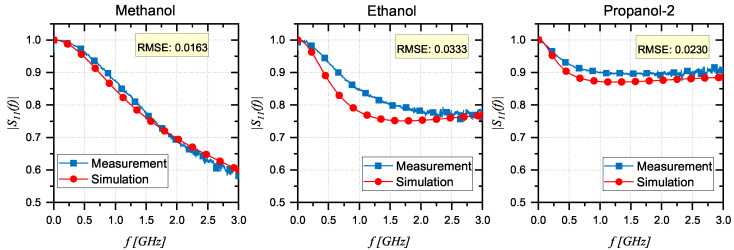
Comparison between the simulated and measured values of the $|S_{11}\left(f\right)|$ on reference liquids: methanol, ethanol, and propanol-2.

Figure 4 presents the comparison between the measurements and simulations in terms of the magnitude of $S_{11}\left(f\right)$ on RDSs after completing the optimization procedure. The results illustrate a good match between the responses of the actual system and the simulated model, and this comparison has been quantitatively assessed using the Root Mean Square Error (RMSE) reported in Figure 4 for each reference liquid. As a figure of merit, the low RMSE values underscore the high fidelity of the simulation in replicating the real behavior of the system. The only notable deviation occurs at 1 GHz with ethanol, likely due to its molecular resonance at microwave frequencies, which leads to increased dielectric losses. However, this is not a concern for biocomposites, as they exhibit different molecular structures and interactions with electromagnetic fields, resulting in significantly lower dielectric losses at this frequency [47,48,49]. The results successfully confirmed the validation of the simulated model and its potential for practical applications. In this way, it is possible to adopt the simulated model for the estimation of the dielectric properties of CBs through the minimization procedure given in Section 3.2.

### 3.2. Final Dielectric Permittivity Estimation

The final procedure consists of adjusting the simulation parameters in terms of real and imaginary parts of dielectric permittivity to align the simulated model’s output with empirical data. The detailed procedure for extrapolating the dielectric properties of CBs through EM simulations can be described in three steps, as shown in Figure 3 and summarized as follows:

(a) *Reflectometric measurement:* the procedure initiates by conducting a reflectometric measurement on the CB under test. This involves using the developed experimental setup to measure how much of an EM signal is reflected by the CB in terms of $|S_{11}\left(f\right)|$.
(b) *Minimization procedure:* the empirical data collected from the reflectometric measurement are then input into the EM simulator and serve as a target point for the simulation. The main goal here is to reduce the discrepancy between the simulated reflection scattering parameter, denoted as $S_{11,mod}\left(f\right)$ and the actual measured value, $S_{11,real}\left(f\right)$. To accomplish this, the procedure lies in fine-tuning the dielectric parameters within the EM simulator until a satisfactory match between the simulation and measurement is obtained. Specifically, discrete points of $\varepsilon^{\prime }$ and $\varepsilon^{\prime \prime }$ were carefully selected to minimize computational resources without risking loss of information. Subsequently, these values were parameterized and subjected to an iterative process that continued until the difference between the simulation and measurement fell within an acceptable range.
(c) *Extrapolation of dielectric properties:* after the simulation results closely mirror the empirical measurements, the adjusted dielectric properties within the simulation are considered to reflect the real properties of the CB under test. After achieving a good match, the next step involved interpolating the values of $\varepsilon^{\prime }$ and $\varepsilon^{\prime \prime }$. This interpolation is used to reconstruct the frequency-dependent trend of both the real and imaginary parts of the permittivity.

## 4. Experimental Results

**Figure 5:**
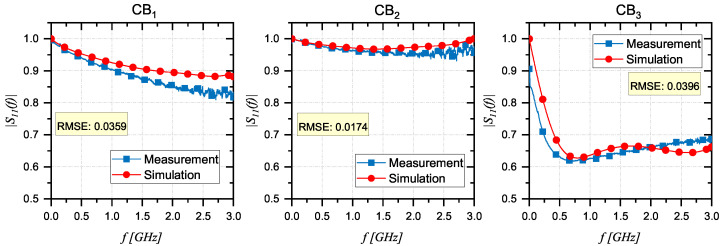
Comparative analysis of $|S_{11,real}|$ and $|S_{11,mo}|$ parameters: a visualization of the optimized match between experimental measurements and simulations for three CB prototypes.

As previously stated, the proposed method was adopted to retrieve the dielectric properties of the three developed innovative CB prototypes ($CB_{1},CB_{2}$, and $CB_{3}$), described in detail in Section 2.3. For each CB, a comparison is presented in Figure 5 between the $|S_{11,real}|$, measured using the experimental setup, and the modeled or simulated $|S_{11,mod}|$ after the minimization process. This comparison reveals the optimum match between the two curves achieved by the optimizer through the parameterization of dielectric properties during the simulation.

**Figure 6:**
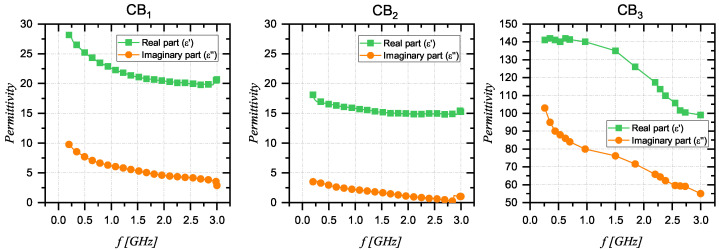
Dielectric properties in terms of $\varepsilon^{\prime }\left(f\right)$ and $\varepsilon^{\prime \prime }\left(f\right)$ for three CB prototypes extrapolated after the minimization procedure.

After the minimization procedure, the final dielectric data in terms of $\varepsilon^{\prime }\left(f\right)$ and $\varepsilon^{\prime \prime }\left(f\right)$ were extrapolated. The obtained results for each CB under test are reported in Figure 6. As a matter of fact, Figure 6 is a direct result of the innovative proposed optimization process described in Section 3.2 and applied to the data presented in Figure 5. As can be observed from the graph, prototypes $CB_{1}$ and $CB_{2}$ exhibit closely related dielectric properties. In contrast, prototype $CB_{3}$ demonstrates a markedly different behavior, with significantly higher permittivity values. The increase in dielectric permittivity in both real and imaginary parts can be explained by considering the effect of sonication on the distribution of expanded graphite fibers in the CB [50]. The energy provided by ultrasound allows for the separation of graphite structures, which increases the surface area and contact points, facilitating the formation of a continuous conductive network [51]. This enhanced structure promotes more effective electron transport within the material.

This leads to an increase in dielectric permittivity, as permittivity is closely related to a material’s polarizability [52]. Additionally, a better dispersion of graphite fibers can reduce the formation of agglomerates, which could act as barriers to polarization and, thus, reduce permittivity [53]. Based on the obtained findings, the proposed approach demonstrates the potential to effectively sense the dielectric properties of thin and flexible CBs, offering several advantages in terms of accuracy, versatility, velocity, and low costs. Another aspect to underline is the efficiency of the proposed method in the analysis of very thin CB materials without damaging them or altering their properties.

## 5. Conclusions

This work has introduced an effective approach for sensing the dielectric properties of CBs using MR technique in combination with a minimization procedure based on a model that accurately simulates the EM response of CBs. This model was fundamental in comparing the measured reflection scattering parameter $S_{11,real}\left(f\right)$ with the simulated one $S_{11,mod}\left(f\right)$, allowing for a precise extrapolation of the dielectric properties. The method’s robustness was proven through its application to three distinct ultra-thin CBs, showing its ability to accurately determine their dielectric properties. The advantages of this technique are manifold. It is non-invasive, avoiding any potential alteration or damage to the sample, which is particularly beneficial for ultra-thin films. Additionally, the method is cost-effective and versatile, suitable for a wide range of CB types and applications. The findings of this research hold significant promise for the fields of flexible electronics and bio-sensing, offering a reliable and efficient tool for sensing CB properties.

## Data Availability

The raw data supporting the conclusions of this article will be made available by the authors on request.

## Abbreviations

The following abbreviations are used in this manuscript:

MDPI	Multidisciplinary Digital Publishing Institute
DOAJ	Directory of open access journals
CBs	Conductive biocomposites
MR	Microwave Reflectometry
m-VNA	Miniaturized Vector Network Analyzer
SE	Sensing element
MUT	Material under test
EM	Electromagnetic
EC	Ethyl cellulose
CF	Carbon fiber
EG	Expanded graphite
EIS	Electrical impedance spectroscopy
spm	Scanning probe microscopy (SPM)
AFM	Atomic force microscopy (AFM)
DSC	Differential Scanning Calorimetry
TGA	Thermogravimetric Analysis
